# Plasma Bicarbonate as a Determinant of Fluid-Induced Acid–Base Changes in Postoperative Critically Ill Patients: A Retrospective Analysis

**DOI:** 10.3390/jcm15051703

**Published:** 2026-02-24

**Authors:** Francesco Zadek, Davide Ottolina, Luca Zazzeron, Matteo Nafi, Jessica Bastreghi, Lucia Gandini, Thomas Langer, Pietro Caironi

**Affiliations:** 1 Department of Medicine and Surgery, University of Milan-Bicocca, 20900 Monza, Italy; francesco.zadek@unimib.it (F.Z.); thomas.langer@unimib.it (T.L.); 2 UO Anestesia e Rianimazione, Ospedale di Saronno, ASST Valle Olona, 21047 Saronno, Italy; davide.ottolina@asst-valleolona.it; 3 Department of Anesthesia, Critical Care and Pain Medicine, Massachusetts General Hospital, Boston, MA 02114, USA; lzazzeron@mgb.org; 4 Department of Anesthesia and Intensive Care Medicine, IRCCS Multimedica, 20099 Sesto San Giovanni, Italy; matteo.nafi@multimedica.it; 5 Department of Anesthesia and Intensive Care Medicine, ASST FBF-Sacco, 20157 Milan, Italy; jessica.bastreghi@asst-fbf-sacco.it; 6 Dipartimento di Emergenza Urgenza, Fondazione IRCCS San Gerardo dei Tintori, 20900 Monza, Italy; lucia.gandini@irccs-sangerardo.it; 7 Department of Anesthesia and Intensive Care Medicine, Niguarda Ca’ Granda, 20162 Milan, Italy; 8 Department of Oncology, University of Turin, 10124 Turin, Italy; 9 Department of Acute Brain and Cardiovascular Injury, Istituto di Ricerche Farmacologiche Mario Negri IRCCS, 20256 Milan, Italy

**Keywords:** fluid therapy, crystalloids, acid–base, balanced solutions

## Abstract

**Background**: Intravenous fluids modify acid–base balance by changing plasma strong ion difference (SID_PL_) and total non-volatile weak acids. Experimental data suggest that pre-infusion plasma bicarbonate (HCO_3_^−^) may further modulate these effects. We tested this hypothesis in a large cohort of postoperative ICU patients receiving intravenous fluids. **Methods**: We retrospectively analyzed all-consecutive post-operative ICU admissions over a 21-month period who received fluid therapy. Fluid inputs/outputs, plasma electrolytes, and arterial blood gases were collected from admission to 9:00 A.M. of postoperative day one. Average SID of infused fluids (SID_INF_) was calculated, and SID_PL_ and standard base excess variations (ΔSBE) were assessed. Patients were stratified by SID_INF_ tertiles (low, <41.0 mEq/L; medium, 41.2–54.6 mEq/L; high, ≥55.0 mEq/L), median pre-infusion HCO_3_^−^ (24.3 [22.4–26.3] mmol/L), and tertiles of SID_INF_-HCO_3_^−^ difference. **Results**: Among 650 admissions, 641 were included (83% elective surgery). Pre-infusion acid–base was, as average, within normal ranges. Total infused volume averaged 2327 ± 1111 mL. Across SID_INF_ tertiles, ΔSBE increased from 1.2 ± 3.4 to 3.0 ± 3.0 and 3.4 ± 3.0 mmol/L (*p* < 0.001), paralleled by ΔSID_PL_ rise (0.6 ± 2.3, 1.3 ± 2.4 and 1.4 ± 2.3 mEq/L, respectively; *p* = 0.004). For any given SID_INF_, patients with lower pre-infusion HCO_3_^−^ showed greater ΔSBE and ΔSID_PL_ (*p* < 0.001). When analyzed by tertiles of SID_INF_-HCO_3_^−^ difference, ΔSBE rose from 1.0 ± 3.2 to 2.7 ± 2.9 and 4.0 ± 3.0 mmol/L (*p* < 0.001), with amplified effects at higher infused volume (>2500 mL). **Conclusions**: In postoperative ICU patients, fluid-induced acid–base changes are largely driven by SID_INF_-HCO_3_^−^ difference, supporting individualized fluid selection based on baseline HCO_3_^−^.

## 1. Introduction

Fluid therapy, i.e., intravenous administration of fluids such as crystalloids, is one of the cornerstones of supportive therapy in critically ill patients during cardiovascular and organ dysfunction [1,2]. Its application is widely implemented for patient volume replacement, fluid maintenance, and intravenous drug administration both in the perioperative setting [3] and, more generally, during hospitalization [4]. In the last decade, evidence has indicated that the type of administered fluid may affect outcomes [5,6,7]. Indeed, the use of balanced solutions has been recently suggested as the preferred types of crystalloids in critically ill patients by clinical practice guidelines, as opposed to isotonic saline [7,8].

Despite its necessity, administration of crystalloids, especially when applied in large volumes, can induce hemodilution and can affect plasma electrolyte concentrations. Consequently, among the potential side effects of crystalloids observed in hospitalized patients, alterations of electrolyte concentration and acid–base equilibrium have been often observed [9,10]. The development of hyperchloremic metabolic acidosis, a representative example, after large volume replacement with isotonic saline during major surgery [11], in trauma patients [12], during kidney transplantation [13,14], and more recently in ketoacidosis [15] has been frequently reported. Of note, the introduction of balanced solutions has aimed at limiting (or avoiding) such potential iatrogenic harm [16].

According to the traditional physical-chemical approach to acid–base equilibrium introduced by Peter Stewart [17], the main determinants of acid–base alterations during intravenous fluid administration are two [18]: (1) the variation of plasma strong ion difference (SID_PL_), as defined by the difference between plasma concentrations of strong cations (mainly Na^+^, K^+^, Mg^2+^, and Ca^2+^) and strong anions (mainly Cl^−^), and related to the administration of fluid volume plus electrolytes; and (2) the reduction of the total concentration of non-volatile weak acids (A_TOT_, mainly albumin and phosphate) occurring during fluid administration, and resulting from dilution, since commonly employed fluids (crystalloids and synthetic colloids) do not contain albumin or phosphate [19,20,21]. Indeed, whereas the two aforementioned determinants are both involved in acid–base variation during fluid resuscitation in the case of fluid maintenance only SID_PL_ variation will be at play, as in theory no A_TOT_ variation should occur. In addition, over a given time period, SID_PL_ variation should result from the average SID of all intravenously administered fluids (SID_INF_) and their volume [20].

Experimental studies performed in both in vitro [21] and in vivo [22] settings have hypothesized the key role of pre-infusion plasma bicarbonate (HCO_3_^−^) concentration, i.e., the plasma concentration of HCO_3_^−^ at the start of fluid administration, as a third determinant of the effects of fluid therapy on acid–base. At constant arterial partial pressure of carbon dioxide (PaCO_2_), pH variations after fluid replacement with crystalloids will be predictable by the difference between the SID of the crystalloids infused and the pre-infusion HCO_3_^−^ concentration [18,21]. Therefore, given a constant SID of infused crystalloids, a different variation of acid–base will be observed depending on the plasma HCO_3_^−^ concentration at the start of crystalloid administration.

To test whether this principle may hold true also in hospitalized patients, receiving a relatively large amount of intravenous fluids in a pragmatic context, without controlling minute ventilation and PaCO_2_, and having a variable pre-infusion HCO_3_^−^ concentration, we set out a retrospective analysis of a large cohort of critically ill patients admitted to a postoperative intensive care unit (ICU). Here, we aim to describe the effects of intravenous fluid therapy on acid–base from ICU admission to the first postoperative day, and to evaluate the potential impact of plasma HCO_3_^−^ concentration on these effects.

## 2. Materials and Methods

This is a single-center retrospective study conducted at Fondazione IRCCS Ca’ Granda—Ospedale Maggiore Policlinico of Milan. The study was approved by the local Institutional Review Board (#3148, on 30 November 2007, Ethical Committee of Fondazione IRCCS Ca’ Granda—Ospedale Maggiore Policlinico, Milan, Italy), which granted access to clinical data recorded on patient clinical charts and waived patient consent based upon the observational nature of the study. De-identification methodology was applied to patient records before analyses.

### 2.1. Study Population

All consecutive patients admitted to the postoperative ICU of our institution between April 2006 and December 2007 were enrolled in the study. Patients were studied from their ICU admission to 9:00 a.m. of the first postoperative day. We excluded patients who did not receive any fluid therapy (i.e., fluid administration other than fluids as medication diluent) during the study period. No further exclusion criteria were included.

### 2.2. Data Collection

Data on patients’ demographics, comorbidities, and medications, as well as reason for admission and type of surgery were recorded. To evaluate the effect of fluid therapy on acid–base balance, the volume and the type of any fluids administered during study period (including crystalloids, colloids, blood products, and crystalloids as drug diluent and intravenous line patency maintenance, i.e., “fluid creep” [23]) were retrieved from the clinical chart and recorded in a dedicated database. Similarly, information regarding all sources of fluid loss (i.e., urine output, aspirated gastric content, and drainage fluids) was obtained. Both at the time of ICU admission and at the end of the study period, data on blood gas analyses (GEM Premier3000, Werfen Instrumentation Laboratory, Milan, Italy), plasma electrolyte concentrations, and laboratory exams, including renal, liver, coagulation, and metabolic parameters, were obtained (COBAS c 702; Roche Diagnostics GmbH, Mannheim, Germany) and included in the database. Information on hemodynamic and respiratory parameters during the study period was also included.

### 2.3. Definitions

Arterial standard base excess (SBE) was calculated as follows [24]:

(1) $$\begin{array}{c} SBE\;(mmol/L)=[({HCO}_{3}^{-}-24.4)+\left(2.3\times Hb/3+7.7\right)\\ \times \left(pH-7.4\right)]\times (1-0.023\times Hb/3)] \end{array}$$

where HCO_3_^−^ denotes plasma bicarbonate concentration expressed in mmol/L, and Hb the hemoglobin concentration expressed in mmol/L.

Plasma strong ion difference (SID_PL_) was calculated as follows [17]:

(2) $${SID}_{PL}\;(mEq/L)=[{Na}^{+}+K^{+}+{Mg}^{2+}+{Ca}^{2+}]-[{Cl}^{-}+{lactate}^{-}]$$

where Na^+^, K^+^, Mg^2+^, Ca^2+^, Cl^−^, and lactate^−^ respectively denote sodium, potassium, magnesium, calcium, chloride, and lactate, all expressed in mEq/L. Ca^2+^ was estimated from total calcium content [25] (see Supplementary Material for further details).

To evaluate the impact of fluid therapy on acid–base balance and plasma electrolyte concentrations, we calculated the difference (∆) in SBE and SID_PL_ assessed at the end of the study (9:00 a.m. of the first postoperative day) and at ICU admission. The in vivo strong ion difference (SID_S_) of each infused solution was calculated as the charge difference between all strong (i.e., fully dissociated in water medium) cations and strong anions, assuming complete metabolism of organic anions (Table S1 of the Supplementary Material for further details).

The average SID infused over the study period (SID_INF_) was calculated as follows:

(3) $${SID}_{INF}\;(mEq/L)=\frac{\Sigma \;({SID}_{S}\times V)}{\Sigma \;V}$$

where SID_S_ denotes the in vivo SID in mEq/L of each fluid, and V the volume of each fluid administered during the study period, as expressed in liters.

The plasma concentrations of non-volatile weak acids (A_TOT_) and their dissociated form (A^−^) were respectively estimated, at ICU admission, as follows [26,27]:

(4) $$\begin{array}{c} A^{-}(mEq/L)=albumin\times 10\times \left(0.123\times arterial\;pH-0.631\right)\\ +P\times 0.309\times (arterial\;pH-0.469) \end{array}$$

where albumin is expressed as g/dL, and P indicates the plasmatic concentration of phosphates in mg/dL.

(5) $$A_{TOT}\;(mmol/L)=A^{-}\times (1+10^{\left(arterial\;pH-6.8\right)})/(10^{\left(arterial\;pH-6.8\right)})$$

The total volume of crystalloid solutions was calculated as the sum of the volume administered as Rehydrating-III, normal saline, Ringer’s lactate, Darrow’s solution, and dextrose 5% (Table S1 of the Supplementary Material). The total colloid solution volume was calculated as the sum of the volume administered as gelatin and 6% hydroxyethyl-starch. Finally, the total volume of blood products administered was estimated assuming standard volumes, as follows: 50 mL per vial of 20% albumin, 300 mL per unit of red blood cells, 250 mL per unit of fresh frozen plasma, and 430 mL per pool of platelets, respectively. Net fluid balance over the study period was calculated as the difference between total fluid administered, including fluid creep, and the total output volume. Overall, no imputation was applied for potentially missing data.

### 2.4. Study Subgroups

To evaluate the effect of the fluid therapy on acid–base balance, we first divided the study population according to the tertiles of SID_INF_, based on its frequency distribution (Figure S1 of the Supplementary Material): low-SID_INF_, medium-SID_INF_, and high-SID_INF_. Thereafter, to explore the impact of pre-infusion plasma HCO_3_^−^ concentrations on the effect of fluid therapy on acid–base at similar SID_INF_, the study population was further stratified according to the median value of plasma HCO_3_^−^ concentration at ICU admission, i.e., before fluid therapy start (Figure S2 of the Supplementary Material). In addition, to investigate the combined effect of SID_INF_ and pre-infusion HCO_3_^−^ concentration on acid–base variations, we calculated the difference between SID_INF_ and plasma HCO_3_^−^ concentration at ICU admission for each patient and divided the study population into tertiles of this index. Finally, to assess the impact of infused volume, patients were arbitrarily divided according to the total amount of fluid infused during the study period: less than 1700 mL, from 1700 mL to 2500 mL, greater than 2500 mL.

### 2.5. Statistical Analysis

Data are presented as mean ± standard deviation, median [interquartile range], or frequency (percentage), as appropriate. Normality distribution was tested by applying the Shapiro–Wilk test. Comparison of demographic and baseline characteristics was performed by Student’s *t*-test, the Mann–Whitney rank sum test, as well as the chi-square test or Fisher’s exact test as appropriate. Variations of acid–base variables and electrolyte concentrations over time were analyzed by applying one-way and two-way analyses of variance (ANOVA) with Holm–Sidak’s correction; in case of non-normally distributed data, Kruskal–Wallis ANOVA on ranks with Dunn’s correction was applied. Multivariable linear regression models were employed to assess the independent association between fluid-related variables and acid–base variation (ΔSBE), while adjusting for potential confounders [28]. Models were also used to compare the relative informational contribution of SID_INF_-HCO_3_^−^ difference and in vivo infused SID (SID_INF_). Covariates were selected a priori based on their clinical relevance to the primary outcome [29]. Model comparison was performed using Akaike’s and Bayesian information criteria (AIC and BIC), with lower values indicating stronger support [30,31]. A *p*-value < 0.05 was considered statistically significant. Analyses were performed using Stata statistical software (Stata Statistical Software 19.5; StataCorp, College Station, TX, USA) and SigmaPlot 15.0 (Systat Software, San Jose, CA, USA). The Strengthening the Reporting of Observational Studies in Epidemiology checklist [32] was employed (see Supplementary Material).

## 3. Results

### 3.1. Study Population

During the study period, a total of 650 patients were admitted to ICU. Nine patients were excluded as not receiving any source of fluid therapy, leaving therefore 641 patients for the analysis. Patients were divided according to the tertiles of SID_INF_ as follows: low-SID_INF_ subgroup, with a SID_INF_ < 41.0 mEq/L (21.7 ± 12.9 mEq/L); medium-SID_INF_ subgroup, with a SID_INF_ between 41.2 and 54.9 mEq/L (49.1 ± 4.2); and high-SID_INF_ subgroup, with a SID_INF_ ≥ 55.0 mEq/L (55.2 ± 1.2 mEq/L). Demographic and clinical characteristics of the overall study population and study subgroups by tertile of SID_INF_ are summarized in Table 1. As shown, low-SID_INF_ patients had a higher age, a lower BMI, and a relatively higher prevalence of chronic kidney disease as compared to other groups (*p* < 0.01 for all, Table 1). Overall, the majority of patients (83%) were admitted to ICU after elective surgery, whereas 12% and 5% were respectively admitted after emergency surgery and for medical reasons (Table S2).

### 3.2. Acid–Base Balance and Plasma Electrolyte Concentration at ICU Admission

At ICU admission, arterial blood gas analysis of the overall study population revealed, as average, normal values of acid–base balance: pH 7.41 ± 0.06, PaCO_2_ 38 ± 6 mmHg, SBE 0.2 ± 4.1 mEq/L, and SID_PL_ 39.2 ± 2.8 mEq/L (Table 2). Despite minor differences in acid–base parameters and partially in electrolyte concentrations between SID_INF_ subgroups, on average all the assessed parameters were within physiological ranges.

### 3.3. Effect of SID_INF_ on Acid–Base Balance

**Figure 1:**
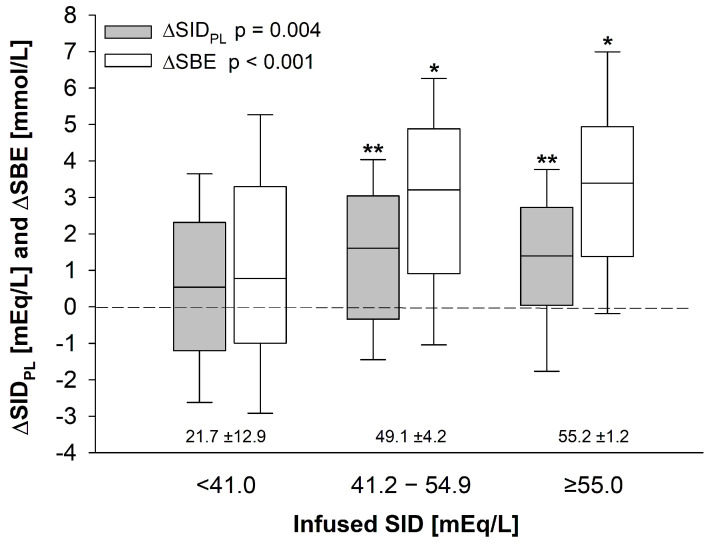
Effect of the average SID infused (SID_INF_) on SBE and plasma SID (SID_PL_) variation during study period by tertile of the average SID infused in the overall study population. Data are presented as median, 25–75, and 10–90 interquartile range. *p*-values refer to one-way analysis of variance (ANOVA) with post-hoc all pairwise multiple comparison procedures (Holm–Sidak correction methods). * *p* < 0.05; ** *p* < 0.001.

To evaluate the effects of the infused fluid therapy, we compared acid–base variations during the study period across subgroups. Low-SID_INF_ patients showed no significant changes in arterial pH, whereas arterial pH increased significantly in the medium- and high-SID_INF_ subgroups (*p* < 0.05 for both), paralleled by a significant increase in both SID_PL_ and SBE (*p* < 0.01 for both, Figure 1; Table S3 of the Supplementary Material). Overall, higher SID_INF_ values were associated with greater increase in SID_PL_ and SBE (*p* = 0.004 and *p* < 0.001, respectively; Figure 1). Of note, no significant changes in PaCO_2_ were observed (Table 3).

### 3.4. Effect of Pre-Infusion HCO_3_^−^ Concentration on Fluid-Induced Acid–Base Changes

**Figure 2:**
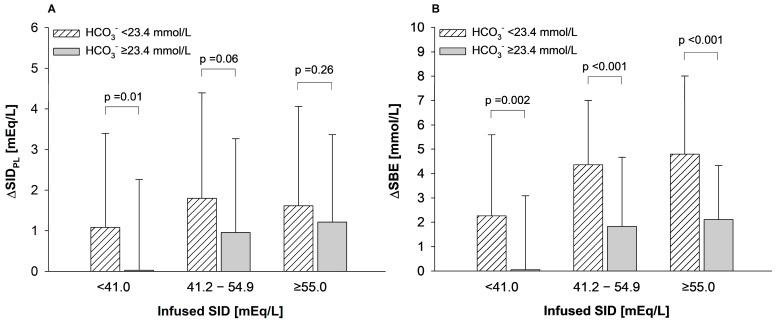
Effect of pre-infusion HCO_3_^−^ concentration on plasma SID (**A**) and SBE (**B**) variation during study period by tertile of the average SID infused in the overall study population. Patients in each SID_INF_ subgroup were divided according to the median value of pre-infusion HCO_3_^−^ concentration observed in the entire study population. Data are presented as mean ± standard deviation. Analysis was performed by two-way analysis of variance (ANOVA), with post-hoc all pairwise multiple comparison procedures (Holm–Sidak correction methods).

To further explore the potential additional role of pre-infusion HCO_3_^−^ concentration in modulating acid–base changes associated with fluid therapy, we stratified each SID_INF_ subgroup by the median value of pre-infusion HCO_3_^−^ concentration of the overall study population (24.3 mmol/L, [22.4–26.2]), ranging from 11.4 mmol/L to 40.1 mmol/L (Figure S2 of the Supplementary Material). As shown, within the same SID_INF_ subgroup (i.e., for the same SID_INF_ infused), patients with lower pre-infusion HCO_3_^−^ values showed a greater increase in SID_PL_ and SBE as compared to patients with higher pre-infusion HCO_3_^−^ values (two-way ANOVA, *p* < 0.01 for HCO_3_^−^ values, for both; Figure 2A,B; Table S4 of the Supplementary Material). The effect was consistent across SID_INF_ subgroups, with no significant interaction for either ∆SID_PL_ (*p* = 0.47) or ∆SBE (*p* = 0.76). To finally confirm the role of the difference between SID_INF_ and pre-infusion HCO_3_^−^ concentration as a determinant of acid–base variation associated with fluid therapy, we analyzed the overall study population as divided by tertiles of SID_INF_-HCO_3_^−^ difference.

**Figure 3:**
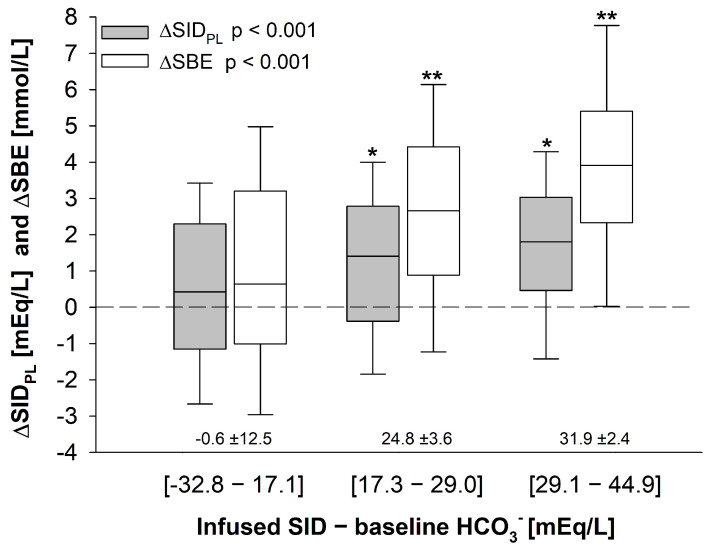
Fluid-induced acid–base variation during study period in relation to the difference between the average SID infused and pre-infusion HCO_3_^−^ concentration in the overall study population. Data are presented as median, 25–75, and 10–90 interquartile range. *p*-values refer to one-way analysis of variance (ANOVA) with post-hoc all pairwise multiple comparison procedures (Holm–Sidak correction methods). * *p* < 0.05 and ** *p* < 0.001 vs. patients within the first tertile of SID_INF_-HCO_3_.

As shown (Figure 3), both ∆SID_PL_ and ∆SBE significantly increased along SID_INF_-HCO_3_^−^ tertiles (*p* < 0.001 for both), indicating a more pronounced effect as SID_INF_-HCO_3_^−^ difference increased. Of note, when both variables were jointly examined in multivariable linear regression models adjusting for clinically relevant confounders, the SID_INF_-HCO_3_^−^ difference appeared to describe SBE variations more accurately than SID_INF_ alone, as supported by AIC and BIC values (Table S5 of the Supplementary Material). Importantly, the model including both variables yielded lower AIC/BIC values compared with the model including SID_INF_ alone, indicating a more informative description of fluid-induced acid–base variations.

### 3.5. Volume Effect

**Figure 4:**
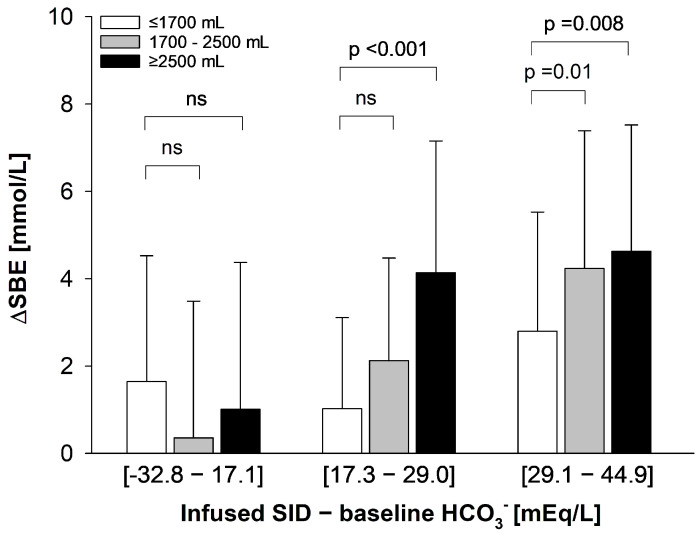
Impact of total fluid volume infused during study period on acid–base variation (SBE) by tertile of the difference between the average SID_INF_ and the pre-infusion HCO_3_^−^ concentration in the overall study population. Data are presented as mean ± standard deviation. Analysis was performed by two-way analysis of variance (ANOVA), with post-hoc all pairwise multiple comparison procedures Holm–Sidak correction methods).

Finally, to assess the impact of the infused volume on the observed acid–base variations of both SID_INF_ and pre-infusion HCO_3_^−^ concentration, we stratified each SID_INF_-HCO_3_^−^ subgroup by the total amount of fluid infused during the study period: less than 1700 mL, from 1700 mL to 2500 mL, and greater than 2500 mL. As shown (Figure 4), whereas no difference in SBE was observed in the subgroup with lower SID_INF_-HCO_3_^−^ difference by total amount of fluid infused, both in the medium and higher SID_INF_-HCO_3_^−^ difference subgroups, ∆SBE progressively increased with the increase in the total amount of fluid infused, indicating that infusion volume may act as a key amplifier of the acid–base variation observed as associated with fluid therapy.

## 4. Discussion

In the current study, performed in a large cohort of patients admitted to a postoperative ICU and receiving intravenous fluid therapy, we observed that: (1) the greater the average in vivo SID of the total amount of infused fluids, the greater the observed increment in SID_PL_, SBE, and pH; (2) for the same average in vivo SID infused, patients with lower pre-infusion HCO_3_^−^ manifest a greater increment in SID_PL_ and SBE than patients with higher pre-infusion HCO_3_^−^ values; and (3) the difference between SID_INF_ and pre-infusion HCO_3_^−^ concentration appears to describe fluid-induced acid–base variation more accurately than the average in vivo SID infused (i.e., SID_INF_) alone.

To the best of our knowledge, these are the first findings clearly confirming that fluid-induced acid–base variations are highly dependent on the difference between the in vivo SID of the infused fluids and the pre-infusion concentration of HCO_3_^−^ in patients with uncontrolled ventilation, i.e., in an “open system”.

Of note, from a methodological standpoint, it is important to highlight that being in a system open to variable alveolar ventilation and PaCO_2_, the proper outcome variable to assess fluid-induced acid–base variation is not pH [21,22], but rather base excess (SBE). Indeed, SBE variation mainly describes the “metabolic” component of acid–base variation, as related to variation in SID_PL_ and/or A_TOT_, whereas pH variation will depend on the combined effect of both the “respiratory” (PCO_2_) and the “metabolic” (SID_PL_ and A_TOT_) components.

In our study population, we observed that the administered fluid therapy led to an increase in SBE in each of the SID_INF_ subgroups, causing a slight degree of metabolic alkalosis (Figure 1 and Table 3). Previous experimental studies performed in both in vitro and in vivo settings have indicated a value of about 24 mEq/L as the in vivo SID_INF_ of the theoretical “balanced” crystalloid [19,21,22], i.e., a crystalloid solution that in the context of fluid therapy/resuscitation (associated with some extent of hemodilution) does not modify acid–base, simultaneously balancing the acidotic effect of SID_PL_ reduction and the alkalotic effect of A_TOT_ dilution. The reason for such a value stems from the normal concentration of HCO_3_^−^ (≈24 mEq/L). In fact, as the charged gap of SID is filled up only by the dissociated portion of A_TOT_, i.e., A^−^, and HCO_3_^−^, based on the principle of electroneutrality and to maintain constant acid–base, any dilution (reduction) of A^−^ induced by fluid administration must be paralleled by a consensual reduction of SID_PL_, so as to maintain unchanged HCO_3_^−^ concentration [21]. Therefore, the exact in vivo SID_INF_ of the theoretical “balanced” solution should be equal to ~24 mEq/L and not 40–42 mEq/L. Accordingly, the administration of a fluid with an in vivo SID_INF_ lower than 24 mEq/L will induce acidosis, as occurs with normal saline (in-vivo SID_INF_ = 0 mEq/L), whereas the administration of a fluid with an in vivo SID_INF_ higher than 24 mEq/L will induce alkalosis, as occurs with crystalloids such as Rehydrating-III or Plasmalyte (in vivo SID_INF_ = 55 or 50 mEq/L, respectively) [18].

However, there are three further main conditions that must be satisfied for this principle to hold true: (A) the organic anions normally included in balanced solutions to fill the charged space of the in vitro SID must be metabolized to HCO_3_^−^; (B) the pre-infusion SID_PL_ must be normal and equal to ~40–42 mEq/L; and (C) the pre-infusion HCO_3_^−^ concentration must be within the normal range, i.e., ~24 mEq/L. Indeed, in our study population, pre-infusion baseline values of SID_PL_ and HCO_3_^−^ were respectively 39 ± 3 mEq/L and 24 ± 3 mmol/L. The average in vivo SID_INF_ infused of the three SID_INF_ subgroups was respectively 21.7 ± 12.9 mEq/L (low-SID_INF_ subgroup), 49.1 ± 4.2 mEq/L (medium-SID_INF_ subgroup), and 55.2 ± 1.2 (high-SID_INF_ subgroup), and the vast majority of patients (82% of the study population) received an average in vivo SID_INF_ higher than 24 mEq/L. Of note, in the low-SID_INF_ subgroup in which in vivo SID_INF_ approximated 24 mEq/L, ΔSBE as average was close to 0 mmol/L (1.2 ± 3.5 mmol/L). In contrast, ΔSBE in the medium- and high-SID_INF_ subgroups equaled 3.0 ± 3.0 mmol/L and 3.4 ± 3.0 mmol/L, respectively.

Despite the onset of metabolic alkalosis after fluid administration, no compensatory respiratory response was observed once patients were spontaneously breathing following a short period of mechanical ventilation. Although we cannot exclude the role of possible confounders (such as the effectiveness of analgesic therapies, albeit standardized, or additional unmeasured stimuli affecting spontaneous breathing patterns), our data may suggest a transient disequilibrium between ΔSID_PL_ and cerebrospinal fluid (CSF) SID, which plays a key role in the control of breathing [33,34]. Indeed, unlike during respiratory acid–base alterations, during metabolic modifications induced by SID_PL_ changes, plasma ions do not passively and rapidly diffuse through the blood–brain and blood–CSF barriers, causing therefore a transient dissociation between plasma and CSF electrolyte composition [35,36,37,38]. Experimental and clinical data [39,40] suggest that an equilibrium between the two compartments may presumably be achieved within 12 to 48 h, depending on the cause, the specific mechanisms, and the rate of plasma (extracellular) acid–base change. Of note, in our cohort, the time frame of acid–base variation observed equaled 18 [15–20] hours. It is therefore conceivable that acid–base alterations induced by fluid therapy within a relatively short period of time (12–24 h) may not be associated with respiratory compensation, if enabled, depending on the rate and the extent at which the alteration develops. Notably, the duration of controlled or supported mechanical ventilation was limited to few hours of the total duration of the study period (4 [3–5] of 18 [15–20] hours, Table 3), and patients remained spontaneously breathing after extubation.

In our study population, pre-infusion HCO_3_^−^ values ranged from 11.4 to 40.1 (Figure S2 of the Supplementary Material). For the same average SID_INF_ infused during the study period, patients with different pre-infusion HCO_3_^−^ values showed markedly different acid–base alterations. Moreover, the SID_INF_-HCO_3_^−^ difference appeared to be a more informative descriptor of fluid-induced acid–base changes than the average SID_INF_ alone (Figure 3 and Supplementary Material), even after adjustments for confounders. Although based on a retrospective analysis, taken together our findings provide proof-of-concept evidence for a role of pre-infusion HCO_3_^−^ concentration as an additional determinant of fluid-induced acid–base alterations, as also reflected by the stronger informational support of the complete multivariable regression model. In addition, the effect of SID_INF_-HCO_3_^−^ on acid–base appeared to be dependent on the total volume administered (see Figure 4, *p* < 0.001 for interaction), indicating that the larger the total fluid volume administered, the higher the ΔSBE for the same SID_INF_-HCO_3_^−^ difference applied. In contrast, no interaction was observed between total fluid volume administered and ΔSBE within the low-SID_INF_ subgroup (average SID_INF_-HCO_3_^−^ of −0.7 ± 12.8 mEq/L), similar to what has been described in vitro by Gattinoni’s group [21]: independently of the total fluid volume infused, no variation of SBE will be observed as long as the SID_INF_ of the infused fluid is roughly identical to the pre-infusion HCO_3_^−^ concentration.

What are the possible clinical implications of our findings? The main message of our study may be straightforward and clinically relevant: whenever evaluating the effect of fluid therapy on acid–base, we should always consider not only the specific type of fluid (i.e., SID_INF_) but also the specific plasma HCO_3_^−^ concentration that our patient presents at the time of fluid therapy prescription. In patients with COPD, or hypercapnic ARDS, clinically presenting, for instance, with a plasma HCO_3_^−^ of ~40 mEq/L, even a crystalloid solution with in vivo SID_INF_ of 24 mEq/L such as Ringer’s lactate or acetate will add metabolic acidosis (SBE reduction) to the clinical picture, as the SID_INF_-HCO_3_^−^ difference will be negative (SID_INF_ < HCO_3_^−^). Similarly, in patients with chronic respiratory alkalosis, such as during pregnancy, or with metabolic acidosis, clinically presenting with a plasma HCO_3_^−^ of ~15 mEq/L, a crystalloid solution such as Ringer lactate or acetate will add metabolic alkalosis (SBE increase), as SID_INF_ will be higher than HCO_3_^−^. In addition, according to this principle, a crystalloid solution such as Plasmalyte, characterized by an in vivo SID_INF_ of ~50 mEq/L, will always induce some degree of metabolic alkalosis as long as the patient’s pre-infusion plasma HCO_3_^−^ is below ~50 mEq/L. In the end, the ideal “balanced” fluid, or crystalloid, does not necessarily imply an in vivo SID_INF_ of ~24 mEq/L [20], but rather it should be tailored to the specific patient plasma HCO_3_^−^ concentration.

Our study also has some limitations. First, it is based on a single-center retrospective analysis with an old dataset (2006–2007) and may therefore be subject to uncontrolled biases and unmeasured confounding variables. Nonetheless, the accuracy of data retrieval, the observational design, and the largely unchanged fluid management during the immediate postoperative period support the robustness of our analysis, albeit inherently limiting causal inference. Second, the range of the average in vivo SID_INF_ values considered in the analysis is not extensive, as the vast majority of participants received an average SID_INF_ ~55 mEq/L (as largely depending on Rehydrating-III crystalloid infusion). Third, no direct data were available on A_TOT_ variation, as albumin and phosphate assessment was not consistently available at the end of the study. However, data on Hb variation between groups, suggesting a similar intravascular dilution effect, and the interaction between total fluid volume and ΔSBE within SID_INF_-HCO_3_^−^ subgroups support the validity of our hypothesis, even in the absence of ΔA_TOT_ assessment. Fourth, no data on urine electrolyte excretion were collected, thereby precluding the evaluation of the contribution of the renal system to fluid-induced acid–base variations. Similarly, we were unable to accurately assess the potential effect of diuretics on fluid-induced acid–base alterations, as detailed information on type, timing, and dosing was not consistently available. However, in our cohort, diuretic use per se did not appear to play a relevant role, as suggested by the multivariable analyses. Finally, our analysis did not included any patient-centered outcomes, thereby limiting the evaluation of the potential clinical relevance of our findings.

## 5. Conclusions

In conclusion, our findings clearly highlighted the importance of considering patient pre-infusion HCO_3_^−^ concentration as a key determinant of fluid-induced acid–base alterations, in parallel with in vivo SID of the fluid employed and the extent of A_TOT_ dilution. Further studies are warranted to prospective validate these findings, especially in the context of different HCO_3_^−^ concentrations, and to evaluate their potential impact on patient-centered outcomes.

## Figures and Tables

**Table 1:** Demographic and clinical characteristics of the overall study population according to the tertile distribution of the average SID infused (SID_INF_).

		Infused SID			
Variable	All Population n = 641	Low-SID_INF_ (<41.0) n = 212	Medium-SID_INF_ (41.2–54.9) n = 160	High-SID_INF_ (≥55.0) n = 269	*p*-Value
**Parameters at admission to ICU**					
Age, year	63 ± 16	65 ± 15	64 ± 16	60 ± 17	0.008
Female sex, n. (%)	272 (42)	81 (38)	73 (46)	117 (44)	0.31
BMI, kg/m^2^	27.1 ± 7.2	25.8 ± 6.1	25.4 ± 5.6	29.3 ± 8.2	<0.001
**Cause of admission to ICU**					
Elective surgical cases, n. (%)	534 (83)	162 (76)	130 (81)	242 (90)	<0.001
Emergency surgical cases, n. (%)	80 (12)	33 (16)	24 (15)	23 (9)	
Medical cases, n. (%)	27 (5)	17 (8)	6 (4)	4 (1)	
**Medication history**					
Diuretics, n. (%)	191 (30)	71 (34)	50 (31)	70 (26)	0.16
Calcium channel blocker, n. (%)	120 (19)	42 (20)	30 (19)	48 (18)	0.84
Angiotensin receptor binders, n. (%)	54 (9)	15 (7)	14 (9)	25 (9)	0.69
Angiotensin-converting enzyme inhibitors, n. (%)	174 (27)	48 (23)	45 (28)	81 (30)	0.19
Beta-blockers, n. (%)	176 (28)	64 (31)	36 (23)	76 (28)	0.22
**Comorbidities**					
Hypertension, n. (%)	332 (52)	117 (55)	81 (51)	134 (50)	0.48
Chronic obstructive pulmonary disease, n. (%)	77 (12)	28 (13)	26 (16)	23 (9)	0.05
Chronic heart failure, n. (%)	37 (6)	17 (8)	7 (4)	13 (5)	0.23
Diabetes, n. (%)	126 (20)	41 (19)	31(19)	54 (20)	0.96
Chronic kidney disease, n. (%)	58 (9)	37 (18)	12 (8)	9 (3)	<0.001
Creatinine clearance, mL/min/1.73 m^2^	96 ± 39	83 ± 43	104 ± 43	101 ± 30	<0.001

BMI denotes body mass index; FiO_2_ inspiratory fraction of oxygen; ICU intensive care unit; SID strong ion difference. Data are presented as mean ± standard deviation, or n (%), as appropriate. Data on creatinine clearance were available for 608 patients.

**Table 2:** Hemodynamics, respiratory, and acid–base parameters of the overall study population at study entry according to the tertile distribution of the average SID infused (SID_INF_).

		Infused SID			
	All Population n = 641	Low-SID_INF_ (<41.0) n = 212	Medium-SID_INF_ (41.2–54.9) n = 160	High-SID_INF_ (≥55.0) n = 269	*p*-Value
**Hemodynamic and oxygenation**					
Heart rate, beats/min	75 ± 18	77 ± 20	75 ± 18	73 ± 16	0.19
Mean arterial pressure, mmHg	91 ± 15	88 ± 15	90 ± 14	94 ± 14	<0.001
Hemoglobin, g/dL	11.9 ± 1.9	11.7 ± 2.0	11.5 ± 1.8	12.3 ± 1.6	<0.001
Mechanically ventilated, n. (%)	523 (81)	174 (82)	146 (91)	197 (73)	<0.001
PaO_2_, mmHg	161 ± 59	158 ± 56	167 ± 60	159 ± 60	0.34
FiO_2_, %	50 [45–50]	50 [40–50]	50 [45–50]	50 [45–50]	0.38
**Plasma Acid-Base Parameters**					
pH	7.41 ± 0.06	7.41 ± 0.07	7.43 ± 0.06	7.41 ± 0.06	0.03
PaCO_2_, mmHg	38 ± 6	37 ± 6	38 ± 6	39 ± 6	0.002
HCO_3_^−^, mmol/L	24.4 ± 3.2	23.7 ± 3.5	25.0 ± 3.5	24.5 ± 2.7	0.002
SBE, mmol/L	0.2 ± 4.1	−0.6 ± 4.5	1.1 ± 4.3	0.2 ± 3.5	0.01
Na^+^, mEq/L	139 ± 3	138 ± 4	139 ± 3	139 ± 3	0.002
K^+^, mEq/L	3.9 ± 0.5	4.2 ± 0.6	3.8 ± 0.5	3.8 ± 0.4	<0.001
Ca^2+^, mEq/L	2.3 ± 0.1	2.3 ± 0.1	2.3 ± 0.2	2.4 ± 0.1	0.58
Mg^2+^, mEq/L	1.4 ± 0.2	1.4 ± 0.3	1.4 ± 0.3	1.4 ± 0.2	0.88
Cl^−^, mEq/L	105 ± 4	105 ± 5	106 ± 3	106 ± 3	0.26
Lactate^−^, mEq/L	1.3 ± 1.0	1.4 ± 1.2	1.2 ± 0.9	1.2 ± 0.8	0.17
SID_PL_, mEq/L	39.2 ± 2.8	38.7 ± 3.0	39.4 ± 2.7	39.4 ± 2.6	0.04
A_TOT_, mmol/L	19.0 ± 2.8	18.6 ± 3.0	18.5 ± 3.0	19.5 ± 2.2	<0.001

PaO_2_ denotes arterial partial pressure of oxygen; FiO_2_ inspired oxygen fraction; Na^+^ sodium; K^+^ potassium; Ca^2+^ ionized calcium; Mg^2+^ magnesium Cl^−^ chloride; SID strong ion difference; A_TOT_ total non-volatile weak acids; PaCO_2_ arterial partial pressure of carbon dioxide; HCO_3_^−^ bicarbonate; SBE standard base excess concentrations. Data are presented as mean ±standard deviation, median [interquartile range], or n (%), as appropriate. Data on PaO_2_ and PaCO_2_ were available for 553 patients; data on pH and SBE, for 552 patients; data on HCO_3_^−^, for 549; data on Ca^2+^, for 531; data on plasma SID for 506; and data on A_TOT_, for 494 patients.

**Table 3:** Fluid administered and acid–base variation during the study period in the overall study population according to the tertile distribution of the average SID infused (SID_INF_).

		Infused SID			
Variable	All Population n = 641	Low-SID_INF_ (<41.0) n = 212	Medium-SID_INF_ (41.2–54.9) n = 160	High-SID_INF_ (≥55.0) n = 269	*p*-Value
Study period, hours	18 [15–20]	18 [14–20]	18 [15–20]	18 [15–20]	0.77
Duration of mechanical ventilation, hours	4 [3–5]	4 [3–6]	4 [3–6]	3 [3–4]	0.31
Furosemide administration, n. (%)	134 (21)	61 (29)	30 (19)	43 (16)	0.002
Furosemide dose, mg	20 [10–25]	15.0 [10–30]	20 [10–25]	20 [10–30]	0.21
ΔpH	0.2 ± 0.7	0.00 ± 0.07	0.02 ± 0.06	0.03 ± 0.06	<0.001
ΔPCO_2_, mmHg	1.5 ± 7.1	2.2 ± 8.0	2.2 ± 6.0	0.6 ± 7.0	0.11
ΔHCO_3_^−^, mmol/L	2.0 ± 2.6	1.1 ± 2.4	2.4 ± 2.6	2.4 ± 2.6	<0.001
ΔSBE, mmol/L	2.6 ± 3.3	1.2 ± 3.4	3.0 ± 3.0	3.4 ± 3.0	<0.001
ΔSID_PL_, mEq/L	1.1 ± 2.3	0.6 ± 2.3	1.3 ± 2.4	1.4 ± 2.3	0.004
ΔHb, g/dL	−0.7 ± 1.2	−0.7 ± 1.2	−0.7 ± 1.1	−0.7 ± 1.3	0.26
Total infusions, mL	2327 ± 1111	2583 ± 1411	2612 ± 878	1957 ± 827	<0.001
Crystalloids	2163 ± 971	2323 ± 1213	2338 ± 775	1933 ± 806	<0.001
Colloids	81 ± 200	135 ± 252	147 ± 241	−	<0.001
Blood products	86 ± 207	135 ± 241	127 ± 235	24 ± 130	<0.001
Urine output, mL	1210 ± 799	1206 ± 822	1164 ± 690	1240 ± 842	0.61
Fluid balance, mL	893 ± 1296	1178 ± 1514	1155 ± 1059	506 ± 1156	<0.001
SID_INF_, mEq/L	42.6 ± 16.8	21.7 ± 12.9	49.1 ± 4.2	55.2 ± 1.2	<0.001
SID_INF_—HCO_3_^−^, mEq/L	18.8 ± 15.9	−0.7 ± 12.8	24.0 ± 5.5	30.7 ± 3.1	<0.001

PaCO_2_ denotes arterial partial pressure of carbon dioxide; SID strong ion difference; SBE standard base excess; Hb hemoglobin; and HCO_3_^−^ bicarbonate concentration. Data are presented as mean ± standard deviation, median [interquartile range], or n (%), as appropriate. Data on delta pH, PaCO_2_, and SBE were available for 457 patients; and data on delta SID, for 412 patients. Crystalloid includes the volume administered with Rehydrating-III, normal saline 0.9%, Ringer’s lactate, Darrow solution, and dextrose 5%. Colloid includes the volume administered with gelatin and hydroxyethyl-starch; blood product includes the volume administered with 20% albumin, red blood cells, fresh frozen plasma, and platelets.

## Data Availability

The dataset is available from the corresponding author on reasonable request.

## Supplementary Materials

The following supporting information can be downloaded at: https://www.mdpi.com/article/10.3390/jcm15051703/s1. Table S1. Composition of fluids infused during the study period; Table S2. Cause of admission to ICU; Table S3. Acid–base variations induced by fluid administration during study period in the overall study population according to the tertile distribution of the average SID infused; Table S4. Acid–base variations induced by fluid administration during study period in the overall study population according to the tertile distribution of the average SID infused (SID_INF_); Table S5. Multivariable linear regression models for SBE variations during study period in the overall study population; Figure S1. Frequency distribution of patients according to the average SID infused (SID_INF_) during the study period; Figure S2. Frequency distribution of patients according to the pre-infusion plasma HCO_3_^−^ concentration recorded at study entry; Figure S3. Impact of total fluid volume infused during study period on acid–base variation (SBE) by tertile of the average SID infused (SID_INF_) in the overall study population; STROBE Statement—Checklist.

## Abbreviations

The following abbreviations are used in this manuscript:

A^−^	Dissociated form of total non-volatile weak acids
A_TOT_	Plasma concentration of total non-volatile weak acids
BMI	Body mass index
Ca^2+^	Calcium
Cl^−^	Chloride
FiO_2_	Inspired oxygen fraction
Hb	Hemoglobin
HCO_3_^−^	Plasma bicarbonate concentration
ICU	Intensive care unit
K^+^	Potassium
Ma^2+^	Magnesium
Na^+^	Sodium
P	Phosphate
PaCO_2_	Arterial partial pressure of carbon dioxide
SBE	Standard base excess
SID	Strong ion difference
SID_PL_	Plasma strong ion differences
SID_INF_	Average infused strong ion difference
