# Supplementary material for: Plasma Bicarbonate as a Determinant of Fluid-Induced Acid–Base Changes in Postoperative Critically Ill Patients: A Retrospective Analysis
Source: J Clin Med. 2026 Feb 24;15(5):1703. doi: 10.3390/jcm15051703 (PMC12985604; doi:10.3390/jcm15051703)
Supplement: Supplementary file 1 [file jcm-15-01703-s001.zip › jcm-4071115-supplementary.pdf]

Plasma bicarbonate as a determinant of acid-base variations induced by fluid therapy in postoperative critically ill patients: a retrospective study

Francesco Zadek[1]*, Davide Ottolina[2]*, Luca Zazzeron[3], Matteo Nafi[4], Jessica Bastreghi[5], Lucia Gandini[6], Thomas Langer[1,7], and Pietro Caironi[8]

Online supplementary materials

Supplementary methods

The ionized calcium concentration was calculated by converting the total calcium concentration into ionized calcium according to De Antonio's formula [1]:

$$Ca^{2+}(\frac{mEq}{L}) = 2 \times 0.815 \times CaTot^{0.5}$$

where $CaTot$ expressed as mmol/L.

The estimated glomerular filtrate rate (eGFR) was calculated as follows:

Male gender

$$eGFR = 186 \times (Creatinine^{-1.154}) \times (age^{-0.203})$$

Female gender

$$eGFR = 186 \times (Creatinine^{-1.154}) \times (age^{-0.203}) \times 0.742$$

where creatinine is expressed as mg/dL, and age in years.

Table S1. Composition of fluids infused during the study period.

	Na^+ [mEq/L]	K^+ [mEq/L]	Ca^{++} [mEq/L]	Mg^{++} [mEq/L]	Cl^- [mEq/L]	Lactate [mEq/L]	Acetate [mEq/L]	Citrate [mEq/L]	Dextrose [mmol/L]	Glucose [mEq/L]	Gelatin [g/L]	HES [g/L]	Albumin [g/L]	Octaonate [mEq/L]	SID [mEq/L]
Rehydrating III	140	10	5	3	103	0	47	8	0	0	-	-	-	-	55
Sodium-Chloride 0.9%	154	0	154	0	0	0	0	0	0	0	-	-	-	-	0
Ringer's lactate	130	4	3	0	109	28	0	0	0	0	-	-	-	-	28
Darrow	126	36	0	0	104	52	0	0	0	0	-	-	-	-	52
Dextrose 5%	0	0	0	0	0	0	0	0	260	0	-	-	-	-	0
Gelatin	145	5.1	12.5	0	145	0	0	0	0	0	35	-	-	-	17.6
HES	154	0	0	0	154	0	0	0	0	0	-	100	-	-	0
Albumin 20%	130	0	0	0	77	0	0	0	0	0	-	-	200	16	53
Red Blood cells	119 ±4	45 ±6	1 ±0	0 ±0	100 ±3	26 ±3	0	0	0	323 ±50	-	-	0 ±0	-	38 ±2
Fresh frozen plasma	170 ±1	3 ±0	7 ±1	1 ±0	73 ±2	2 ±1	0	0	0	370 ±11	-	-	37±2	-	100 ±2
Platelets	172 ±2	2 ±0	3 ±0	1 ±0	91 ±1	4 ±0	0	0	0	144 ±6	-	-	14 ±1	-	79 ±1

Na^+ denotes sodium, K^+ potassium, Ca^{++} ionized calcium, Mg^{++} magnesium Cl^- chloride, HES hydroxy-ethyl starches, and SID strong ion difference. Data on blood-derived components were obtained from Langer T et al. [2].

Table S2. Cause of admission to ICU

Type of surgery	All population<br>n = 641	Infused SID			p value
		Low-SID$_{INF}$<br>(<41.0)<br>n = 212	***Medium-SID$_{INF}$***<br>(41.2-54.8)<br>n = 160	***High-SID$_{INF}$***<br>(≥55.0)<br>n = 269	
General surgery, n. (%)	187 (29)	65 (31)	53 (33)	69 (26)	0.14
Thoracic surgery, n. (%)	158 (26)	46 (24)	44 (29)	68 (26)	0.42
Urologic surgery, n. (%)	76 (12)	34 (16)	15 (9)	27 (10)	0.08
Hepato-biliary surgery, n. (%)	67 (11)	20 (10)	22 (14)	25 (9)	0.30
Bariatric surgery, n. (%)	59 (10)	11 (6)	7 (5)	41 (16)	<0.001
Vascular surgery, n. (%)	40 (7)	13 (7)	8 (5)	19 (7)	0.71
Other surgeries, n. (%)	27 (4)	6 (3)	5 (3)	16 (6)	0.21

P values refer to chi-square or Fisher's exact analysis, as appropriate.

Table S3. Acid-base variations induced by fluid administration during study period in the overall study population according to the tertile distribution of the average SID infused.

		Infused SID						
	**All population** n = 641	***Low-SID$_{INF}$*** (<41.0) n = 212	***Medium-SID$_{INF}$*** (41.2 − 54.9) n = 160	***High-SID$_{INF}$*** (≥55.0) n = 269	**Infused SID p value**	**Groups p value**	**Time p value**	**Interaction p value**
pH pre	7.41 ±0.06	7.41 ±0.07	7.43 ±0.06	7.41 ±0.06	0.03	0.005	<0.001	<0.001
pH post	7.43 ±0.05	7.41 ±0.06	7.44 ±0.04	7.44 ±0.03	<0.001			
PCO_2 pre, mmHg	38 ±6	37 ±6	38 ±6	39 ±6	0.002	0.13	<0.001	0.07
PCO_2 post, mmHg	40 ±6	39 ±8	40 ±5	40 ±4	0.39			
HCO_3^- pre, mmol/L	24.4 ±3.2	23.7 ±3.5	25.0 ±3.5	24.5 ±2.7	0.002	<0.001	<0.001	<0.001
HCO_3^- post, mmol/L	26.3 ±3.1	25.0 ±3.5	27.2 ±3.0	26.8 ±2.5	<0.001			
SID_{PL} pre, mEq/L	39.2 ±2.8	38.7 ±3.0	39.4 ±2.7	39.4 ±2.6	0.04	0.002	<0.001	0.006
SID_{PL} post, mEq/L	40.3 ±2.3	39.5 ±2.7	40.6 ±2.3	40.7 ±1.9	<0.001			
SBE pre, mmol/L	0.2 ±4.1	-0.6 ±4.5	1.1 ±4.3	0.2 ±3.5	0.01	<0.001	<0.001	<0.001
SBE post, mmol/L	2.7 ±3.7	0.9 ±4.2	3.7 ±3.4	3.4 ±2.7	<0.001			
Hb pre, g/dL	11.9 ±1.9	11.7 ±2.0	11.5 ±1.8	12.3 ±1.6	<0.001	<0.001	<0.001	0.78
Hb post, g/dL	11.2 ±1.7	10.9 ±1.7	10.8 ±1.6	11.7 ±1.7	<0.001			

$PaCO_2$ denotes arterial partial pressure of carbon dioxide; HCO_3^- bicarbonate concentration; SBE standard base excess; SID strong ion difference; and Hb hemoglobin concentration. Data are presented as mean ±standard deviation. p values refer to two-way analysis of variance (ANOVA) with one factor for repetition (time and SID_{INF} subgroups). Post hoc all-pairwise multiple-comparison procedures were performed using the Holm-Sidak correction.

Table S4. Acid-base variations induced by fluid administration during study period in the overall study population according to the tertile distribution of the average SID infused (SID_{INF}).

	Low-SID_{INF} (<41.0)		***Medium-SID_{INF}*** (41.2 − 54.9)		***High-SID_{INF}*** (≥55.0)		
	Low Bic	High Bic	Low Bic	High Bic	Low Bic	High Bic	p value
	(N = 75)	(N = 71)	(N = 56)	(N = 65)	(N = 86)	(N = 101)	
pH pre	7.39 ±0.06	7.44 ±0.06	7.40 ±0.06	7.44 ±0.05	7.39 ±0.06	7.42 ±0.06	0.27
pH post	7.40 ±0.05	7.43 ±0.07	7.43 ±0.04	7.45 ±0.04	7.44 ±0.03	7.44 ±0.03	0.56
Delta pH	0.01 ±0.07	-0.02 ±0.07	0.03 ±0.06	0.01 ±0.05	0.04 ±0.06	0.03 ±0.06	0.57
	(N = 75)	(N = 72)	(N = 56)	(N = 65)	(N = 86)	(N = 101)	
PCO_2 pre, mmHg	35.1 ±4.9	39.4 ±5.5	36.0 ±4.7	39.8 ±5.8	36.5 ±4.4	41.0 ±6.0	0.33
PCO_2 post, mmHg	37.1 ±4.4	41.7 ±9.6	38.7 ±4.2	41.6 ±5.4	38.4 ±4.0	40.6 ±4.4	0.39
Delta PCO_2, mmHg	2.1 ±6.0	2.3 ±9.7	2.7 ±6.5	1.8 ±5.6	1.8 ±6.2	-0.4 ±7.6	0.36
	(N = 75)	(N = 72)	(N = 56)	(N = 65)	(N = 86)	(N = 101)	
HCO_3^- pre, mmol/L	21.2 ±2.4	26.7 ±2.2*	22.1 ±1.5	27.1 ±3.2*	22.2 ±1.7	26.4 ±1.6*	0.03
HCO_3^- post, mmol/L	23.1 ±2.8	27.0 ±2.9*	25.6 ±2.7	28.6 ±2.7*	25.8 ±2.3	27.7 ±2.3*	0.002
Delta HCO_3^-, mmol/L	1.9 ±2.5	0.3 ±2.1*	3.5 ±2.2	1.4 ±2.5*	3.6 ±2.6	1.3 ±2.1*	0.35

Table S4. *Continued*

	(N = 68)	(N = 63)	(N = 46)	(N = 61)	(N = 79)	(N = 92)	
SID pre, mEq/L	37.7 ±2.9	40.1 ±2.4	38.1 ±2.7	40.0 ±2.4	38.6 ±2.7	40.0 ±2.0	0.64
SID post, mEq/L	38.7 ±3.0	40.2 ±2.0	39.9 ±2.0	41.0 ±2.3	40.2 ±1.8	41.2 ±1.8	0.14
Delta SID, mEq/L	1.1 ±2.3	0.0 ±2.2	1.8 ±2.6	1.0 ±2.3	1.6 ±2.4	1.2 ±2.2	0.47
	(N = 75)	(N = 71)	(N = 56)	(N = 65)	(N = 86)	(N = 101)	
SBE pre, mmol/L	-3.5 ±3.2	3.1 ±2.8*	-2.4 ±2.3	3.5 ±3.6*	-2.5 ±2.6	2.3 ±2.6*	0.01
SBE post, mmol/L	-1.2 ±3.6	3.2 ±3.5*	1.9 ±3.1	5.3 ±2.9*	2.3 ±2.5	4.4 ±2.5*	0.002
Delta SBE, mmol/L	2.3 ±3.3	0.0 ±3.0*	4.4 ±2.6	1.8 ±2.8*	4.8 ±3.2	2.1 ±2.2*	0.76
	N = 94)	(N = 75)	(N = 64)	(N = 79)	(N = 98)	(N = 114)	
Hb pre, g/dL	11.6 ±1.8	11.7 ±2.0	11.7 ±1.8	11.3 ±1.9	12.2 ±1.5	12.4 ±1.7	0.20
Hb post, g/dL	10.8 ±1.5	11.1 ±1.8	10.9 ±1.6	10.8 ±1.7	11.4 ±1.4	11.8 ±1.9	0.33
Delta Hb , g/dL	-0.8 ±1.2	-0.6 ±1.1	-0.8 ±1.3	-0.5 ±1.0	-0.8 ±0.9	-0.6 ±1.5	0.63

$PaCO_2$ denotes arterial partial pressure of carbon dioxide; HCO_3^- bicarbonate concentration; SBE standard base excess; SID strong ion difference; and Hb hemoglobin concentration. Data are presented as mean ±standard deviation. p values refer to the interaction value of two-way analysis of variance (ANOVA). * $p<0.05$ vs *Low-Bicarbonate group* (post-hoc Holm-Sidak correction methods).

Table S5. Multivariable linear regression models for SBE variations during study period in the overall study population.

Variable	***Model 1*** SID_{INF}	***Model 2*** SID_{INF}-HCO_3^-	***Model 3*** SIDINF + SID_{INF}-HCO_3^-
	β (95% CI)	β (95% CI)	β (95% CI)
SID_{INF}, mEq/L	0.065 (0.045 / 0.085)***	——	-0.438 (-0.536 / -0.339)***
SID_{INF}-HCO_3, mEq/L	——	0.080 (0.061 / 0.099)***	0.510 (0.412 / 0.609)***
SID_{PL} at study entry, mEq/L	-0.343 (-0.454 / -0.233)***	-0.302 (-0.409 / -0.195)***	-0.042 (-0.156 / 0.073)
Creatinine Clearance, ml/min/1.73 m^2	-0.004 (-0.012 / 0.005)	-0.003 (-0.012 / 0.005)	0.004 (-0.004 / 0.012)
Chronic Kidney Disease (yes/no)	-0.204 (-1.395 / 0.986)	-0.167 (-1.320 / 0.987)	-0.787 (-1.854 / 0.281)
Diuretics (yes/no)	-0.415 (-1.062 / 0.231)	-0.319 (-0.948 / 0.310)	-0.075 (-0.655 / 0.504)
Models parameters			
Adjusted R^2	0.17	0.21	0.33
AIC	2032	2009	1940
BIC	2056	2034	1968

SID_{INF} denotes average strong ion difference of the infused fluids, HCO_3^- pre-infusion bicarbonate concentration, SIDPL plasma strong ion difference, β regression coefficient, 95% CI the 95% Confidence Interval, R^2 the squared R of the multivariable model, AIC Akaike Information Criterion, and BIC Bayesian Information Criterion. ***P< 0.01. Multivariable analyses were performed using generalized linear models with Gaussian family and identity link; model assumptions were assessed and found to be satisfied. Covariates were selected a priori based on their clinical relevance to the outcome. β indicates the regression coefficient, representing the adjusted change in the outcome associated with a one-unit increase in the corresponding predictor.

Supplementary Figures

Figure S1. Frequency distribution of patients according to the average SID infused (SID_{INF}) during the study period.

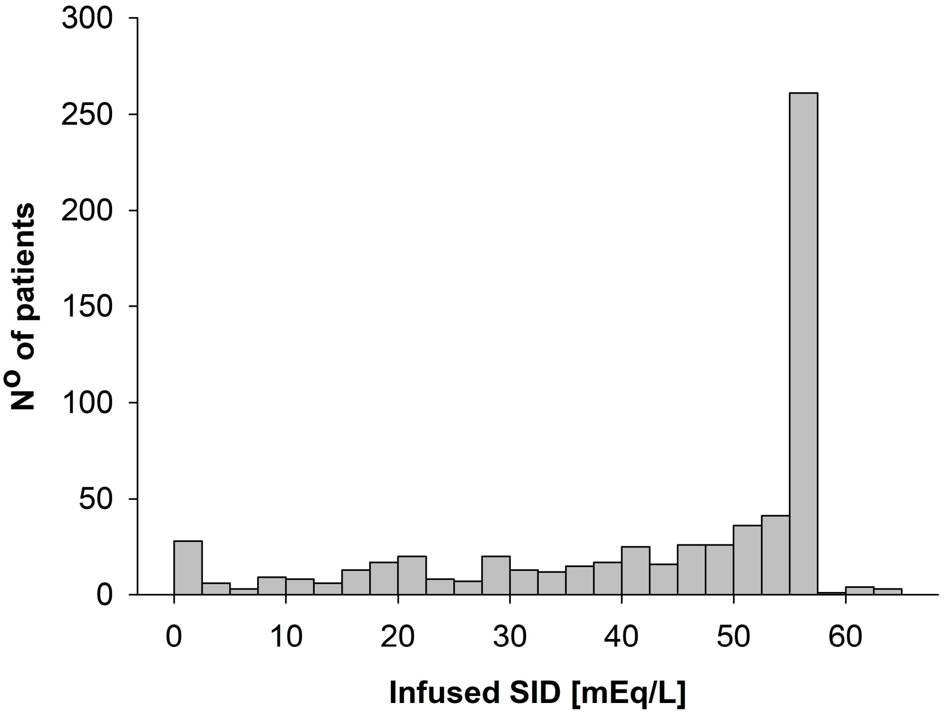

For each patient, the average SID_{INF} was calculated considering all the sources of fluid administered during the study period (see method section of the main manuscript for further details).

Figure S2. Frequency distribution of patients according to the pre-infusion plasma HCO_3^- concentration recorded at study entry.

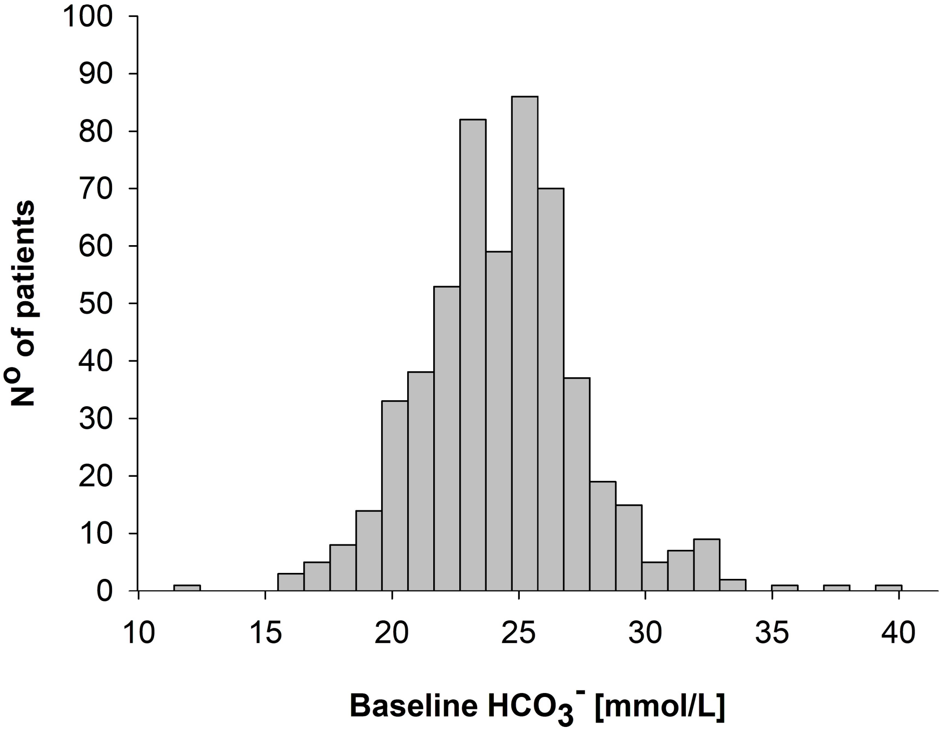

Figure S3. Impact of total fluid volume infused during study period on acid-base variation (SBE) by tertile of the average SID infused (SID_{INF}) in the overall study population.

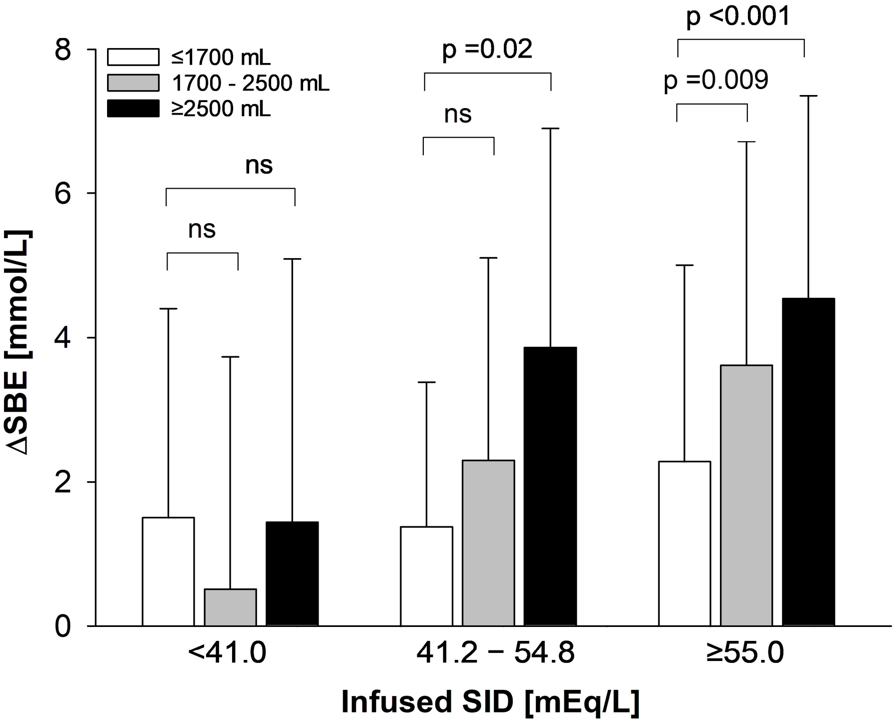

White bars denote patients receiving during the study period a total fluid volume lower than 1700 mL; gray bars denote patients receiving a total fluid volume between 1700 and 2500 mL; black bars denote patients receiving a total fluid volume higher than 2500 mL.Variations refer to the difference for each parameter between end-of-study and entry-study values. Data are presented as mean ± standard deviation. Analysis was performed using a two-way analysis of variance (ANOVA), with post hoc all-pairwise multiple comparisons (Holm-Sidak correction).

References

1. Mateu-De Antonio, J. New Predictive Equations for Serum Ionized Calcium in Hospitalized Patients. *Medical Principles and Practice* 2016, *25*, 219–226, doi:10.1159/000443145.

2. Langer, T.; Ferrari, M.; Zazzeron, L.; Gattinoni, L.; Caironi, P. Effects of Intravenous Solutions on Acid-Base Equilibrium: From Crystalloids to Colloids and Blood Components. *Anaesthesiol Intensive Ther* 2014, *46*, 350–360.

STROBE Statement—checklist of items that should be included in reports of observational studies

	Item No.	Recommendation	Page No.
**Title and abstract**	1	(*a*) Indicate the study's design with a commonly used term in the title or the abstract	1
		(*b*) Provide in the abstract an informative and balanced summary of what was done and what was found	1
**Introduction**			
Background/rationale	2	Explain the scientific background and rationale for the investigation being reported	2
Objectives	3	State specific objectives, including any prespecified hypotheses	2
**Methods**			
Study design	4	Present key elements of study design early in the paper	2
Setting	5	Describe the setting, locations, and relevant dates, including periods of recruitment, exposure, follow-up, and data collection	3
Participants	6	(*a*) *Cohort study*—Give the eligibility criteria, and the sources and methods of selection of participants. Describe methods of follow-up<br>*Case-control study*—Give the eligibility criteria, and the sources and methods of case ascertainment and control selection. Give the rationale for the choice of cases and controls<br>*Cross-sectional study*—Give the eligibility criteria, and the sources and methods of selection of participants	3
		(*b*) *Cohort study*—For matched studies, give matching criteria and number of exposed and unexposed	

		Case-control study—For matched studies, give matching criteria and the number of controls per case	
Variables	7	Clearly define all outcomes, exposures, predictors, potential confounders, and effect modifiers. Give diagnostic criteria, if applicable	3-4
Data sources/ measurement	8*	For each variable of interest, give sources of data and details of methods of assessment (measurement). Describe comparability of assessment methods if there is more than one group	3-4
Bias	9	Describe any efforts to address potential sources of bias	3
Study size	10	Explain how the study size was arrived at	3
Quantitative variables	11	Explain how quantitative variables were handled in the analyses. If applicable, describe which groupings were chosen and why	4
Statistical methods	12	(*a*) Describe all statistical methods, including those used to control for confounding	5
		(*b*) Describe any methods used to examine subgroups and interactions	5
		(*c*) Explain how missing data were addressed	
		(*d*) *Cohort study*—If applicable, explain how loss to follow-up was addressed<br><br>*Case-control study*—If applicable, explain how matching of cases and controls was addressed<br><br>*Cross-sectional study*—If applicable, describe analytical methods taking account of sampling strategy	
		(*e*) Describe any sensitivity analyses	
Participants	13*	(a) Report numbers of individuals at each stage of study—eg numbers potentially eligible, examined for eligibility, confirmed eligible, included in the study, completing follow-up, and analysed	5

		(b) Give reasons for non-participation at each stage	5, 6, 8
		(c) Consider use of a flow diagram	
Descriptive data	14*	(a) Give characteristics of study participants (eg demographic, clinical, social) and information on exposures and potential confounders	5
		(b) Indicate number of participants with missing data for each variable of interest	5, 6, 8
		(c) *Cohort study*—Summarise follow-up time (eg, average and total amount)	3
Outcome data	15*	*Cohort study*—Report numbers of outcome events or summary measures over time	
		Case-control study—Report numbers in each exposure category, or summary measures of exposure	
		Cross-sectional study—Report numbers of outcome events or summary measures	
Main results	16	(*a*) Give unadjusted estimates and, if applicable, confounder-adjusted estimates and their precision (eg, 95% confidence interval). Make clear which confounders were adjusted for and why they were included	7-10
		(*b*) Report category boundaries when continuous variables were categorized	7-10
		(*c*) If relevant, consider translating estimates of relative risk into absolute risk for a meaningful time period	
Other analyses	17	Report other analyses done—eg analyses of subgroups and interactions, and sensitivity analyses	7-9
Key results	18	Summarise key results with reference to study objectives	9
Limitations	19	Discuss limitations of the study, taking into account sources of potential bias or imprecision. Discuss both direction and magnitude of any potential bias	13

Interpretation	20	Give a cautious overall interpretation of results considering objectives, limitations, multiplicity of analyses, results from similar studies, and other relevant evidence	12-13
Generalisability	21	Discuss the generalisability (external validity) of the study results	12-13
**Other information**			
Funding	22	Give the source of funding and the role of the funders for the present study and, if applicable, for the original study on which the present article is based	13
